# Association between the Oxidative Balance Score and Telomere Length from the National Health and Nutrition Examination Survey 1999-2002

**DOI:** 10.1155/2022/1345071

**Published:** 2022-02-09

**Authors:** Wan Zhang, Shu-Fen Peng, Li Chen, Hui-Min Chen, Xue-Er Cheng, Yu-Han Tang

**Affiliations:** Department of Nutrition and Food Hygiene, Hubei Key Laboratory of Food Nutrition and Safety and the Ministry of Education (MOE) Key Lab of Environment and Health, School of Public Health, Tongji Medical College, Huazhong University of Science and Technology, Wuhan 430030, China

## Abstract

**Purpose:**

Leukocyte telomere length (LTL) is an important biomarker of aging. The oxidative balance score (OBS) is used to assess the oxidative stress-related exposures of diet and lifestyle. This study is aimed at exploring if the OBS was associated with LTL.

**Methods:**

3220 adults were included in this study from the National Health and Nutrition Examination Survey (NHANES) 1999-2002. LTL was assayed from leukocyte DNA. Twenty dietary and lifestyle factors were selected to score the OBS. Survey-based multivariable linear regression was conducted to assess the association between the OBS and log-transformed LTL.

**Results:**

The association between the OBS and log-transformed LTL was positive in females but not males. For females, compared with the lowest OBS category as a reference, the multivariable-adjusted beta estimate (95% confidence interval, CI) for the highest OBS category was 0.0701 (0.0205–0.1197) (*p* for trend < 0.01), and the multivariable-adjusted beta estimate (95% CI) of the continuous OBS was 0.0039 (0.0014–0.0065). There was also the gender difference in the correlations of the dietary OBS and the lifestyle OBS with log-transformed LTL.

**Conclusion:**

There was a positive association between the OBS and LTL in females. This result suggested that diet and lifestyle might affect LTL by regulating oxidative balance.

## 1. Introduction

Telomeres are repetitive nucleoprotein regions located at the ends of eukaryotic chromosomes. Researches show that telomere plays an active role in cellular lifespan, genome stability, and chromosome integrity [1]. Since telomere length (TL) is progressively shorter during mitosis over time, it has been proposed to be a biomarker of cell senescence [2, 3]. As TL naturally shortens, cell senescence occurs, life expectancy decreases, and the risk of aging-related diseases increases, such as cancer [4, 5], cardiovascular disease [6], and type 2 diabetes [7].

Beyond chronological age, oxidative stress (OS) is another critical factor that accounts for shorter leukocyte telomere length (LTL) [8, 9]. Telomeres were highly susceptible to OS because of their significant guanine content in human cells [10]. OS refers to the predominance of prooxidants over antioxidants, which increases reactive oxygen and nitrogen species (RONS) [11], potentially leading to damage to lipids, proteins, or DNA. RONS can be the endogenous production of the intracellular mechanism of mitochondrial and cytoplasmic enzyme systems and can be the exogenous production of diet, lifestyle, drugs, and environmental toxins. There is mounting evidence of high intakes of certain nutrients, such as vitamin C [12], vitamin D [13, 14], vitamin E [15], calcium [16, 17], magnesium [18], zinc [19], and selenium [20], protected against OS, while prooxidant factors, including smoking [21], drinking [22], and high iron intake [23], increased RONS production and accelerated OS-related cellular damage in humans. Additionally, there are U-shaped relationships between some nutrients and OS. For example, as a catalytic cofactor of copper-dependent superoxide dismutase and ceruloplasmin, copper is considered an antioxidant since it could increase the activity of these antioxidant enzymes in a proper dose in humans [24]. However, it could catalyze the formation of ROS if chronically overloaded or overexposed [25], and a deficiency in dietary copper could also increase cellular susceptibility to oxidative damage in vivo [26]. As dietary and lifestyle factors were related to the production of RONS, they might affect TL through the oxidation of telomere DNA.

However, studies that evaluated the effects of individual antioxidant or prooxidant exposures on senescence have produced inconsistent results [20, 27–34]. Actually, except for vitamin C [27, 35], which is a well-known strength antioxidant, the associations between other dietary components and LTL were always conflicting, such as carotene, vitamin E, selenium, and iron [20, 27–30]. Similarly, the effects of some lifestyle factors, including drinking [31, 32], physical activity [33], and obesity [34], on LTL were also controversial. One potential explanation for this discrepancy may be that most nutrients and lifestyle factors have a limited independent effect on LTL. Another explanation is that there may be a biological interaction involving multiple prooxidant and antioxidant factors [36, 37]. Therefore, a combined measurement of various prooxidants and antioxidants could be a more accurate indicator of the overall OS.

The oxidation balance score (OBS) is a comprehensive indicator that reflects the overall exposure balance of dietary and lifestyle prooxidants and antioxidants. Generally, a higher OBS indicates a predominance of antioxidants over prooxidants. There were many studies reporting the negative associations between OBS and aging-related diseases, including type 2 diabetes [38], osteoarthritis [39], cardiovascular disease [40], and cancers [41, 42]. Additionally, telomere attrition is associated with increased morbidity and mortality of various age-related diseases [43]. However, no study has yet assessed the association between OBS and LTL, and the relationship between the comprehensive OS-related exposures of diet and lifestyle and TL remains unclear.

Hence, the purpose of the present study was to evaluate the association between the OBS and LTL in NHANES 1999-2002. Based on the available researches, we hypothesized that the OBS was positively associated with LTL.

## 2. Materials and Methods

### 2.1. Study Population

**Figure 1:**
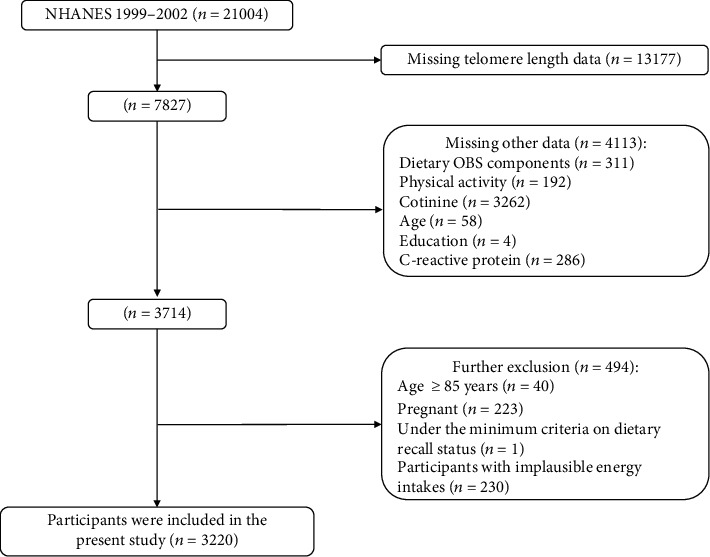
Flowchart depicting the selection strategy.

The National Health and Nutrition Examination Survey (NHANES) is a nationally representative cross-sectional study conducted every two years by the National Center for Health Statistics (NCHS) to assess the health and nutrition status of adults and children in the United States. About 5000 random samples are selected each year using a multistage, stratified probability sampling design. Written informed consent was obtained from all participants in this survey, and details that might disclose the identity of the subjects under study were omitted. Although NHANES has been collecting national data for many decades, only two 2-year survey cycles include information on LTL, 1999–2000 and 2001–2002. Among 21004 participants in the NHANES 1999-2002, only 7827 participants had LTL measurements. And individuals were excluded if (1) they lost data for any of the OBS components or covariates (*n* = 4113), (2) they were ≥85 years old cause all individuals who were aged ≥85 were given the age of 85 by NHANES (*n* = 40), (3) they were pregnant (*n* = 223), (4) the dietary recall status was under the minimum criteria (*n* = 1), and (5) their energy intakes were implausible (male: <800 kcal/d or >4200 kcal/d, female: <500 kcal/d or >3500 kcal/d) (*n* = 230). Finally, a total of 3220 participants were included in the present study (Figure 1).

### 2.2. Data Collection

TL relative to standard reference DNA (T/S ratio) was measured using the quantitative polymerase chain reaction (PCR) method described in detail elsewhere [44, 45]. DNA was isolated from whole blood using the Puregene (D-50K) kit protocol (Gentra Systems, Inc., Minneapolis, Minnesota) and stored at -80°C by the NCHS, and the LTL assay was performed in the laboratory of Dr. Elizabeth Blackburn at the University of California, San Francisco. Each DNA sample was assayed to duplicate wells in a 96-well plate three times on three different days, resulting in six data points. Each assay plate contained 96 control wells with eight control DNA samples (the single-copy gene used as a control was human beta-globin). Control DNA values were used to normalize between-run variability. Sample plates were assayed in groups consisting of three plates, and no two plates were together more than once. The assay runs were excluded from further analysis if runs have invalid control wells ≥ 8 (<1% of runs) or runs have more than 4 control DNA values falling outside 2.5 standard deviations from the mean for all assay runs (<6% of runs). For each sample, the largest and the smallest T/S ratio values were marked as potential outliers; then, the mean and standard deviation of the T/S ratio were calculated excluding the potential outliers. If the absolute value of the log of the ratio between the mean excluding the potential outliers and the value of the potential outlier was greater than 0.4, then the value was marked as an outlier. Any outliers in each sample were identified and excluded from the calculation (<2% of samples). Finally, the mean and standard deviation of the T/S ratio were calculated, and the interassay coefficient of variation was 6.5%.

Both diet and lifestyle contributed to the OBS. In the NHANES, dietary intake data was from 24-hour dietary recall interviews (24HR) which were conducted in the Mobile Examination Center. The types and amounts of foods and beverages consumed during the 24 hours before interviews were collected and recorded in the NHANES computer-assisted dietary interview system. Assessment of dietary intake of nutrients was based on the University of Texas Food Intake Analysis System and the U.S. Department of Agriculture Survey Nutrients Database. Additionally, the nutrient estimates did not include nutrients obtained from dietary supplements or medications. Lifestyle factors related to the OBS in this study were alcohol consumption, smoking, body mass index (BMI), and physical activity. The alcohol consumption information came from the 24HR. To evaluate both active and passive smoking, we used serum cotinine to reflect the smoking. It is a major metabolite of nicotine with a longer half-life than nicotine, could be used to measure the extent of tobacco use and to estimate the extent of exposure to environmental tobacco smoke, and was measured by isotope dilution-high performance liquid chromatography/atmospheric pressure chemical ionization tandem mass spectrometry (ID HPLC-APCI MS/MSID, HPLC: Hewlett-Packard model 1090L, Series II; APCI MS/MS: Mass Spectrometer PE-Sciex API III Triple Quadrupole mass spectrometer). Trained examiners recorded the body measurements of all examinees in the MEC, and BMI was weight divided by height squared (kg/m^2^). The weekly metabolic equivalent (MET) was calculated from the data of the individual-specific leisure-time activities over the past 30 days, and the data was from the household interview.

The covariates contained age, ethnicity, gender, education background, poverty income ratio (PIR), C-reactive protein (CRP), and dietary energy intake. Among covariates, demographic records were taken from household interviews, data of dietary energy intake came from the 24HR, and CRP was quantified using latex-enhanced nephelometry on the Dade Behring Nephelometer II Analyzer System (Dade Behring Diagnostics Inc., Somerville, NJ). We categorized ethnicity as non-Hispanic white, non-Hispanic black, Mexican American, and others. Education background was graded into less than 9th grade, 9-11th grade (includes 12th grade with no diploma), high school grade/general equivalent diploma, some college or associate degree, and college graduate or above. The poverty to income ratio is an index of poverty status that is total family income divided by the poverty threshold. It was graded into three categories based on the analysis guideline: ≤1.3, 1.3-3.5, and >3.5 [46].

### 2.3. OBS

The overall OBS was calculated by summing the points assigned for each component; a higher OBS reflected a predominance of antioxidant exposure. Based on a priori information about the relationship between nutrients or lifestyle factors and OS, sixteen nutrients and four lifestyle factors were screened to calculate the OBS, with five prooxidants and fifteen antioxidants. Most components have been used to calculate OBS at previous [37], and six components were newly selected based on the available data and their association with OS; they were riboflavin [47], niacin [48], vitamin B_6_ [49], vitamin B_12_ [50], magnesium [51], and copper [26]. In addition, smoking was estimated by cotinine as it could measure the extent of both tobacco use and exposure to environmental tobacco smoke.

Table 1 shows the assignment scheme of the OBS components. For alcohol consumption, nondrinkers, nonheavy drinkers (0 to 15 g/d for female and 0 to 30 g/d for male), and heavy drinkers (≥15 g/d for female and ≥30 g/d for male) received 2, 1, and 0 points, respectively. Then, other components were divided into three groups by their sex-specific tertiles. Antioxidants were assigned points from 0 to 2 for groups from tertile 1 to tertile 3, respectively. The point assignment for prooxidants was inverse, with 0 points for the highest tertile and 2 points for the lowest tertile.

The OBS had combined the contributions of both diet and lifestyle. To investigate whether diet or lifestyle factors significantly contributed to the OBS-LTL association, respectively, we calculated a dietary OBS by excluding four lifestyle variables: cotinine, alcohol consumption, BMI, and physical activity from the OBS measures that have been described above and calculated a lifestyle OBS that only included these four variables [52].

### 2.4. Statistical Analysis

Statistical analyses were performed following CDC analytical reporting guidelines for complex NHANES data analysis. We considered the masked variance and used the recommended weighting scheme. In the present study, the individual sample weights were founded on four years of Mobile Examination Center records, as recommended by NHANES. LTL was log-transformed to better approximate a normal distribution. The continuous variables were presented as weighted means ± standard deviations or median (*P*_25_, *P*_75_), and the categorical variables were expressed as unweighted frequencies (weighted percentages). These means and frequencies were generalizable to the American adult population. To test the characteristic differences for variables in different OBS groups (quartiles), the Rau-Scott chi-squared test, the weighted univariate linear regression, and the Kruskal-Wallis test were used for categorical, normal continuous, and nonnormal continuous variables, respectively.

Multivariate linear regression models (SAS SurveyReg procedure) were used to investigate the associations between the OBS and LTL. In general, the OBS was treated as a continuous variable in analyses. To verify the correlation between the OBS and LTL and observe the possibility of a nonlinear relationship between the OBS and LTL, the OBS was converted to a categorical variable by quartile and computed *p* for trend. Four models were applied in the present study. Model one was the crude model without adjustment for any potential confounders. Model two adjusted for age, ethnicity, education level, and PIR. Model three and model four further adjusted for dietary energy intake and CRP, respectively.

The statistical analyses included three main steps. Primarily, the characteristic distribution of data in each gender was described according to the quartile of OBS. Then, the multivariate linear regression models were used to estimate the association between LTL and OBS in males and females, and sensitivity analyses were used to evaluate the robustness of multivariate linear regression results; it was to analyze whether the results would be significantly affected if one component was removed from the total score at one time. Finally, the dietary OBS and the lifestyle OBS were explored if they were associated with LTL, respectively.

All analyses were performed using the statistical software SAS version 9.4 (SAS Institute Inc., Cary, NC). Alpha was set at <0.05 for statistical significance, and all analyses were two-sided.

## 3. Results

### 3.1. Baseline Characteristics

Among the 3220 individuals included in this study, 1697 were males and 1523 were females. Participants had a mean age of 44.5 years (range 20 to 84) in each gender. The baseline characteristics of males and females by quartiles of the OBS are summarized in Tables 2 and 3, respectively. For both males and females, most participants were non-Hispanic white, and compared to those in the lowest OBS quartile, participants in the highest quartile were more likely to be non-Hispanic white. Participants in the highest OBS quartile also tended to have higher educations, higher incomes, higher total energy intakes, and lower CRP. For females, as expected, the log-transformed LTL was higher in the highest OBS quartile when compared with the lowest group (0.08 vs. 0.02, *p* < 0.01). Although there were significant differences in the LTL for OBS quartiles, log-transformed LTL had no significant trend from the low OBS quartile to the high OBS quartile in males.

Supplementary Table 1 presents the distribution of each OBS component according to sex-specific OBS quartiles. Dietary antioxidants and physical activity were higher among participants with a higher OBS. Meanwhile, participants in the higher OBS quartiles were also more likely to have lower levels of alcohol consumption, BMI, and serum cotinine. Contrary to the OBS assignment scheme, intakes of iron and total fat were higher in the upper OBS quartile groups.

### 3.2. Association between the OBS and Log-Transformed LTL

Table 4 shows the association between the OBS with log-transformed LTL (T/S ratio) from multivariate linear regression models. The association was positive in females while not in males. When the OBS was a categorical variable and compared with the lowest OBS category as a reference, the beta estimates (95% confidence intervals, CIs) for the most antioxidative OBS group (quartile 4) of males and females in the full model were -0.0043 (-0.0522–0.0434) and 0.0701 (0.0205–0.1197), respectively. The *p* trend was 0.89 in males and was smaller than 0.01 in females. When treating the OBS as a continuous variable, the beta estimates (95% CIs) of the OBS in the full model were 0.0001 (-0.0020–0.0023) for males and 0.0039 (0.0014–0.0065) for females, compared with the corresponding results after each OBS component was removed from the score one at a time with the original OBS (examined as a continuous variable). Removing any single OBS component did not significantly affect the results of males or females (Supplementary Table 2).

### 3.3. Association between the Dietary OBS/Lifestyle OBS and Log-Transformed LTL

The results of multivariate linear regression analyses to assess the associations of the dietary and lifestyle OBS with log-transformed LTL are presented in Table 5. For females, the multivariable-adjusted beta estimates (95% CIs) for the dietary and lifestyle OBS of females were 0.0034 (0.0009–0.0059) and 0.0147 (0.0032–0.0263), respectively. While the association between the dietary OBS, the lifestyle OBS, and log-transformed LTL were not still statistically significant in males.

## 4. Discussion

In the present study, we explored the association between the OBS and LTL within a large, random, and national sample of American adults. The result showed that the association between the OBS and log-transformed LTL was positive and stable in females but not males. Furthermore, both the dietary OBS and the lifestyle OBS were positively associated with log-transformed LTL only in females as well.

The association between the OBS and the LTL for females was stable and positive. Although there was no direct evidence, several groups had investigated the relations between OBS and aging-related diseases. For example, researchers found that a higher OBS was associated with a lower risk of cancers [41, 42, 53], lumbar spine osteoporosis [54], metabolic syndrome [55], and chronic kidney disease [56]. Additionally, individuals from a biracial American cohort with a higher OBS might have lower all-cause, cancer, and noncancer mortality [57]. Telomere attrition was considered to be positively associated with increased morbidity and mortality of various age-related diseases. Therefore, these researches indirectly supported the positive association between the OBS and the LTL. Besides, similar to previous studies [54–57], the dietary prooxidants (iron and total fat) were higher in the upper OBS quartiles, and this was contrary to the OBS assignment scheme. And this contrary may be driven by the fact that both dietary antioxidants and prooxidants tend to increase as total dietary intake increases and dietary antioxidants contribute more to the OBS than dietary prooxidants.

There was a significant gender difference in the correlations between the OBS and LTL, which was partly consistent with previous researches. Similarly, a positive association between adherence to the Mediterranean diet and TL was observed in a meta-analysis with 13733 participants from 5 countries. However, no association was reported for males in this meta-analysis [58]. Besides, an empirically derived high vegetable dietary pattern was associated with longer LTL in Chinese females but not males [59]. Furthermore, a previous study from NHANES 1999-2002 reported that higher Healthy Eating Index 2010 scores, Alternate Healthy Eating Index, MedDiet scores, and Dietary Approaches to Stop Hypertension scores were each associated with longer telomere length in females but not males [60]. However, in terms of the associations between lifestyle factors and LTL, previous studies usually discussed each lifestyle separately, and the relationships between lifestyle factors and LTL did not always have gender differences. As reported, the BMI was negatively associated with TL in both males and females [61], and the positive association between smoking and TL was always stronger among females [62, 63]. But whether there were gender differences in the associations between physical activity and alcohol consumption, TL was still inconclusive. In the present study, further analyses showed that both the dietary OBS and the lifestyle OBS were significantly associated with LTL in females but not males. These results suggest that the diet and lifestyle with higher antioxidants than prooxidants may protect against telomere shortening in American females, which may further contribute to preventing age-related diseases and reducing the risk of death from it.

The levels of sex hormones may partly contribute to the gender difference. All estrogens have a free phenolic hydroxyl group on the A-ring, which has antioxidant properties and is the sole structural determinant for free radical scavenging [64]. It is likely to interrupt the free radical chain reaction by rapidly donating the hydrogen atom of the phenol hydroxyl group to free radicals [65]. Besides, estrogens also act as potent antioxidants and regulators of antioxidant genes via stimulation of antioxidant enzyme expression and activities, such as superoxide dismutases and glutathione peroxidases [66]. On the contrary, androgens are considered to induce OS because it elevated the metabolic rate that might augment ROS production by increasing oxygen consumption [67]. In addition, in the NHANES 1999-2002, males consumed higher levels of processed meats and sugar-sweetened beverages across quartiles of LTL [60], which were known to induce inflammation, insulin resistance, and OS. The large quantity consumption of these specific foods may strongly weaken the beneficial properties of a high OBS diet on LTL in males. Moreover, research from the Health and Retirement Study (HRS) indicated that the association between smoking and LTL was strongly attenuated among males but not females in cross-sectional analyses [62]. And the attenuation was driven by the fact that male smokers with shorter TL may have been more likely to quit because short TL was correlated with poorer health among males [68]. However, females were less likely to quit to improve physical fitness than were males [69]. Hence, it was speculated that males with shorter TL who quit smoking may have lower serum cotinine than current male smokers with longer TL. This weakened association between cotinine and LTL in males may account for part of the gender difference between the lifestyle OBS and LTL.

There were several strengths associated with the present study. First, the oxidation potential of both diet and lifestyle to LTL was rarely accessed in other studies. Second, a complex, multistage probability sampling design was used to select the sample representative of the civilian noninstitutionalized resident population, so the results were generalizable to all civilian noninstitutionalized resident adults of the United States. Third, this study controlled many confounding factors, including sociodemographic characteristics, dietary energy intake, and CRP. Fourth, the sensitivity analyses provide evidence that the key results of the present study were robust.

The current study also has several limitations. First, the cross-sectional nature of the data makes it difficult to infer causation and strongly weakened the associations between cotinine and LTL in males which partly contributed to the gender difference. Second, the OBS was hard to include all OS-related dietary and lifestyle exposures; many components, such as flavonoids, were limited in the database; moreover, there may be some ambiguous OS-related dietary or lifestyle factors not included. However, the correlation between LTL and the present OBS was stable enough and unlikely to be significantly affected by the nonincluded components. Third, all prooxidants and antioxidants were assumed to linearly correlate with OS without considering the threshold effect of antioxidants. Evidence supported that some antioxidants may exert prooxidant activities in large doses or under certain conditions, such as carotenoids and copper [26, 70]. As for physical activity, although it has been reported to induce increases in ROS production, it appears unlikely that rigorous and prolonged exercise results in an OS level that is detrimental to human health [71]. And in the NHANES 1999-2002 population, adults who participate in high levels of physical activity tend to have longer telomeres [72]. Fourth, the OBS dietary components came from self-report data from 24HR and only one 24HR was used to assess dietary information, so it may be prone to measurement error and biases and may not account for day-to-day variability in diet leading to imprecise estimates. Finally, no OS biomarkers could be used to verify the validity of the OBS for oxidative balance assessment in the present study.

## 5. Conclusion

In conclusion, results from a nationally representative sample of American adults indicated that the OBS was positively associated with LTL in females. It revealed that a higher OBS, which indicated a predominance of antioxidant exposure over prooxidant exposure in diet and lifestyle, was associated with a longer LTL. This finding might suggest a protective effect of adherence to an antioxidative diet and lifestyle on the LTL of American females. These findings require further corroborations from future prospective studies.

## Figures and Tables

**Table 1:** Oxidative balance score assignment scheme.

OBS components	Property	Male			Female		
		0	1	2	0	1	2
Dietary OBS components							
Dietary fiber (g/d)	A	<12.56	12.56-19.70	≥19.70	<10.10	10.10-16.31	≥16.31
Carotene (RE/d)	A	<98.83	98.83-306.25	≥306.25	<98.08	98.08-383.50	≥383.50
Riboflavin (mg/d)	A	<1.79	1.79-2.69	≥2.69	<1.34	1.34-2.02	≥2.02
Niacin (mg/d)	A	<20.65	20.65-29.75	≥29.75	<14.52	14.52-21.86	≥21.86
Vitamin B_6_ (mg/d)	A	<1.59	1.59-2.40	≥2.40	<1.13	1.13-1.77	≥1.77
Total folate (mcg/d)	A	<316.00	316.00-492.00	≥492.00	<251.00	251.00-388.96	≥388.96
Vitamin B_12_ (mcg/d)	A	<3.36	3.36-6.20	≥6.20	<2.22	2.22-4.22	≥4.22
Vitamin C (mg/d)	A	<42.44	42.44-113.21	≥113.21	<38.01	38.01-98.49	≥98.49
Vitamin E (ATE) (mg/d)	A	<5.82	5.82-9.42	≥9.42	<4.53	4.53-7.52	≥7.52
Calcium (mg/d)	A	<646.00	646.00-1072.00	≥1072.00	<499.24	499.24-849.00	≥849.00
Magnesium (mg/d)	A	<257.00	257.00-361.28	≥361.28	<187.00	187.00-283.43	≥283.43
Zinc (mg/d)	A	<9.75	9.75-15.10	≥15.10	<6.73	6.73-10.75	≥10.75
Copper (mg/d)	A	<1.12	1.12-1.57	≥1.57	<0.85	0.85-1.28	≥1.28
Selenium (mcg/d)	A	<94.94	94.94-141.80	≥141.80	<67.79	67.79-99.50	≥99.50
Total fat (g/d)	P	≥69.83	69.83-107.43	<107.43	≥50.98	50.98-75.79	<75.79
Iron (mg/d)	P	≥12.88	12.88-19.17	<19.17	≥9.65	9.65-14.32	<14.32
Lifestyle OBS components							
Physical activity (MET-minute/week)	A	<417.86	417.86-1135.71	≥1135.71	<270.00	270.00-845.71	≥845.71
Alcohol (g/d)	P	≥30	0-30	None	≥15	0-15	None
Body mass index (kg/m^2^)	P	≥25.54	25.54-29.17	<29.17	≥23.74	23.74-28.64	<28.64
Cotinine (ng/mL)	P	≥0.038	0.038-1.13	<1.13	≥0.035	0.035-0.172	<0.172

OBS: oxidative balance score; A: antioxidant; P: prooxidant; RE: retinol equivalent; ATE: alpha-tocopherol equivalent; MET: metabolic equivalent.

**Table 2:** The baseline characteristics of males by quartiles of the OBS: National Health and Nutrition Examination Survey, United States, 1999–2002^a^.

Characteristics	Total (1697)	Q1 (434)	Q2 (382)	Q3 (472)	Q4 (409)	*p*
Age (year)	44.5 ± 15.4	42.8 ± 16.2	44.6 ± 15.0	45.5 ± 15.4	44.8 ± 15.0	0.05
Race/ethnicity						<0.01
Non-Hispanic white (%)	1019	226 (73.20)	223 (78.70)	286 (78.96)	284 (84.07)	
Non-Hispanic black (%)	232	94 (11.81)	51 (5.73)	61 (6.27)	26 (2.85)	
Mexican American (%)	334	92 (7.46)	76 (5.55)	91 (5.23)	75 (5.66)	
Other race (%)	45	8 (2.86)	17 (5.17)	9 (2.45)	11 (3.51)	
Other Hispanic (%)	67	14 (4.67)	15 (4.85)	25 (7.09)	13 (3.91)	
Education						<0.01
Less than 9th grade	132	40 (3.02)	32 (3.92)	45 (3.99)	15 (1.77)	
9-11th grade	237	84 (15.06)	54 (9.85)	55 (10.05)	44 (5.61)	
High school grad/GED	395	114 (25.77)	95 (27.58)	112 (23.67)	74 (17.64)	
Some college or associate degree	436	117 (32.20)	100 (29.22)	126 (29.27)	93 (22.88)	
College graduate or above	497	79 (23.95)	101 (29.43)	134 (33.03)	183 (52.13)	
Family PIR						<0.01
≤1.3	293	98 (17.45)	79 (13.66)	61 (9.4)	55 (9.24)	
1.3-3.5	615	170 (35.80)	134 (33.65)	189 (36.24)	122 (24.77)	
>3.5	789	166 (46.75)	169 (52.69)	222 (54.35)	232 (65.79)	
Energy (kcal)	2477 ± 781	1849 ± 626	2336 ± 667	2636 ± 676	3031 ± 642	<0.01
CRP (mg/dL)	0.14 (0.06, 0.31)	0.16 (0.07, 0.34)	0.15 (0.07, 0.33)	0.14 (0.06, 0.31)	0.13 (0.06, 0.27)	<0.01
Telomere (T/S ratio)	1.03 (0.88, 1.21)	1.04 (0.88, 1.21)	1.04 (0.87, 1.19)	1.00 (0.87, 1.22)	1.03 (0.88, 1.20)	<0.01
Log-transformed telomere (T/S ratio)	0.03 ± 0.24	0.04 ± 0.24	0.02 ± 0.23	0.02 ± 0.24	0.03 ± 0.24	0.62

GED: general equivalent diploma; PIR: the ratio of family income to poverty; CRP: C-reactive protein. ^a^Data were expressed as the means ± standard deviations, median (*P*_25_, *P*_75_), or counts (weighted percentages).

**Table 3:** The baseline characteristics of females by quartiles of the OBS: National Health and Nutrition Examination Survey, United States, 1999–2002^a^.

Characteristics	Total (1523)	Q1 (384)	Q2 (397)	Q3 (347)	Q4 (395)	*p*
Age (year)	44.5 ± 15.5	43.0 ± 15.5	44.7 ± 16.2	45.3 ± 15.3	44.9 ± 14.8	0.07
Race/ethnicity						<0.01
Non-Hispanic white (%)	900	204 (74.22)	213 (74.86)	218 (82.52)	265 (84.40)	
Non-Hispanic black (%)	196	68 (9.27)	69 (9.23)	28 (3.74)	31 (3.59)	
Mexican American (%)	311	79 (4.96)	85 (6.03)	75 (5.12)	72 (4.16)	
Other race (%)	40	11 (3.70)	7 (1.64)	11 (3.92)	11 (3.34)	
Other Hispanic (%)	76	22 (7.85)	23 (8.25)	15 (4.69)	16 (4.51)	
Education						<0.01
Less than 9th grade	119	34 (3.29)	35 (4.01)	31 (3.44)	19 (1.53)	
9-11th grade	184	68 (14.55)	46 (8.55)	36 (8.46)	34 (5.86)	
High school grad/GED	356	110 (30.35)	103 (29.64)	73 (21.23)	70 (17.12)	
Some college or associate degree	498	119 (34.44)	141 (34.81)	118 (34.89)	120 (29.36)	
College graduate or above	366	53 (17.36)	72 (22.68)	89 (31.98)	152 (46.12)	
Family PIR						<0.01
≤1.3	320	107 (25.62)	99 (20.82)	67 (17.53)	47 (8.46)	
1.3-3.5	562	148 (35.72)	135 (29.46)	143 (36.92)	136 (31.12)	
>3.5	641	129 (38.66)	163 (49.72)	137 (45.55)	212 (60.42)	
Energy (kcal)	1818 ± 622	1337 ± 486	1678 ± 501	1985 ± 529	2224 ± 565	<0.01
CRP (mg/dL)	0.22 (0.08, 0.48)	0.24 (0.09, 0.61)	0.22 (0.09, 0.49)	0.22 (0.07, 0.45)	0.18 (0.07, 0.38)	<0.01
Telomere (T/S ratio)	1.04 (0.90, 1.22)	1.03 (0.87, 1.18)	1.03 (0.91, 1.22)	1.03 (0.90, 1.20)	1.08 (0.93, 1.23)	<0.01
Log-transformed telomere (T/S ratio)	0.05 ± 0.24	0.02 ± 0.24	0.05 ± 0.25	0.04 ± 0.23	0.08 ± 0.24	0.02

GED: general equivalent diploma; PIR: the ratio of family income to poverty; CRP: C-reactive protein. ^a^Data were expressed as the means ± standard deviations, median (*P*_25_, *P*_75_), or counts (weighted percentages).

**Table 4:** Beta estimates for the association between the OBS and log-transformed leukocyte telomere (T/S ratio) in each gender^a^.

OBS	Q1	Q2	Q3	Q4	*p* trend	Continue
Males						
Model 1	Ref	-0.0137 (-0.0565–0.0811)	-0.0214 (-0.0686–0.0258)	-0.0104 (-0.0683–0.0258)	0.62	-0.0003 (-0.0026–0.0018)
Model 2	Ref	-0.0079 (-0.0472–0.0314)	-0.0073 (-0.0525–0.0379)	-0.0100 (-0.0559–0.0379)	0.69	-0.0003 (-0.0025–0.0019)
Model 3	Ref	-0.0042 (-0.0444–0.0359)	-0.0009 (-0.0428–0.0409)	-0.0004 (-0.0493–0.0485)	0.97	-0.0003 (-0.0018–0.0025)
Model 4	Ref	-0.0045 (-0.0438–0.0348)	-0.0022 (-0.0431–0.0387)	-0.0043 (-0.0522–0.0434)	0.89	0.0001 (-0.0020–0.0023)
Females						
Model 1	Ref	0.0342 (-0.0140–0.0824)	0.0239 (-0.0140–0.0824)	0.0589 (0.0147–0.1031)	0.02	0.0030 (0.0008–0.0052)
Model 2	Ref	0.0455 (-0.0004–0.0913)	0.0366 (-0.0059–0.0792)	0.0703 (0.0285–0.1121)	<0.01	0.0037 (0.0015–0.0058)
Model 3	Ref	0.0457 (-0.0046–0.0961)	0.0371 (-0.0102–0.0844)	0.0709 (0.0205–0.1214)	0.01	0.0040 (0.0014–0.0066)
Model 4	Ref	0.0448 (-0.0046–0.0941)	0.0369 (-0.0103–0.0842)	0.0701 (0.0205–0.1197)	<0.01	0.0039 (0.0014–0.0065)

^a^Data are expressed as the beta estimates and its 95% confidence intervals. Model 1 was a crude model. Model 2 further adjusted for age, ethnicity, education, poverty index. Model 3 further adjusted for dietary energy intake. Model 4 additionally adjusted for CRP.

**Table 5:** Beta estimates for associations between the dietary/lifestyle OBS and log-transformed leukocyte telomere (T/S ratio)^a^.

OBS	Male	Female
Dietary OBS		
Model 1	-0.0006 (-0.0031–0.0019)	0.0026 (0.0006–0.0047)
Model 2	-0.0005 (-0.0030–0.0019)	0.0032 (0.0011–0.0054)
Model 3	0.0001 (-0.0025–0.0027)	0.0034 (0.0009–0.0059)
Model 4	-0.0000 (-0.0026–0.0025)	0.0034 (0.0009–0.0059)
Lifestyle OBS		
Model 1	0.0037 (-0.0063–0.0137)	0.0131 (-0.0017–0.0279)
Model 2	0.0032 (-0.0054–0.0118)	0.0143 (0.0027–0.0259)
Model 3	0.0030 (-0.0056–0.0116)	0.0151 (0.0034–0.0268)
Model 4	0.0018 (-0.0068–0.0104)	0.0147 (0.0032–0.0263)

^a^Data were expressed as the beta estimates and its 95% confidence intervals. Model 1 was a crude model, including OBS. Model 2 further adjusted for age, ethnicity, education, and poverty index. Model 3 further adjusted for dietary energy intake. Model 4 additionally adjusted for CRP.

## Data Availability

The National Health and Nutrition Examination Survey is an open-access resource, and data are available at https://wwwn.cdc.gov/nchs/nhanes/search/default.aspx.

## Supplementary Materials

Supplementary Materials Supplementary Table 1 presents the individual component distributions of the score by the sex-specific OBS quartiles. Supplementary Table 2 is the result of sensitivity analyses, which was used to evaluate the impact of individual OBS components on the OBS.
