# Supplementary material for: Association between the Oxidative Balance Score and Telomere Length from the National Health and Nutrition Examination Survey 1999-2002
Source: Oxid Med Cell Longev. 2022 Feb 9;2022:1345071. doi: 10.1155/2022/1345071 (PMC8850082; doi:10.1155/2022/1345071)
Supplement: Supplementary Materials — Supplementary Table 1 presents the individual component distributions of the score by the sex-specific OBS quartiles. Supplementary Table 2 is the result of sensitivity analyses, which was used to evaluate the impact of individual OBS components on the OBS. [file 1345071.f1.docx]

# Oxidative Medicine and Cellular Longevity

# Association between the oxidative balance score and telomere length from the National Health and Nutrition Examination Survey 1999-2002

Wan Zhang^1^, Shu‑Fen Peng^1^, Li Chen^1^, Hui‑Min Chen^1^, Xue‑Er Cheng^1^, Yu‑Han Tang^1^

^1^ Department of Nutrition and Food Hygiene, Hubei Key Laboratory of Food Nutrition and Safety and the Ministry of Education (MOE) Key Lab of Environment and Health, School of Public Health, Tongji Medical College, Huazhong University of Science and Technology, Wuhan 430030, China.

Corresponding Author Yuhan Tang, PhD Email: [2015220157@hust.edu.cn](mailto:2015220157@hust.edu.cn)

| Supplementary Table 1: Individual component distributions of the score by sex-specific OBS quartiles^a^ | | | | | |
| --- | --- | --- | --- | --- | --- |
| Characteristics | Total | Q1 | Q2 | Q3 | Q4 |
| Dietary OBS components | | | | | |
| Dietary fiber (g/d) |  |  |  |  |  |
| Male | 17.81 ± 11.05 | 9.69 ± 4.50 | 13.23 ± 5.08 | 18.95 ± 7.91 | 27.85 ± 13.46 |
| Female | 14.54 ± 8.30 | 7.59 ± 3.35 | 11.82 ± 4.96 | 15.73 ± 6.38 | 22.28 ± 9.11 |
| carotene (RE/d) |  |  |  |  |  |
| Male | 439.60 ± 848.08 | 144.09 ± 275.30 | 295.11 ± 526.96 | 454.22 ± 760.51 | 813.43 ± 1261.63 |
| Female | 473.85 ± 809.88 | 224.73 ± 504.32 | 299.69 ± 472.11 | 498.37 ± 750.51 | 847.70 ± 1151.35 |
| Riboflavin (mg/d) |  |  |  |  |  |
| Male | 2.37 ± 1.13 | 1.41 ± 0.53 | 1.99 ± 0.72 | 2.53 ± 0.87 | 3.40 ± 1.18 |
| Female | 1.83 ± 0.88 | 1.06 ± 0.39 | 1.52 ± 0.55 | 2.00 ± 0.65 | 2.65 ± 0.92 |
| Niacin (mg/d) |  |  |  |  |  |
| Male | 26.97 ± 13.22 | 17.44 ± 7.24 | 23.28 ± 8.85 | 27.80 ± 9.25 | 37.77 ± 15.89 |
| Female | 19.47 ± 9.47 | 11.49 ± 4.68 | 16.68 ± 6.21 | 21.88 ± 8.29 | 26.92 ± 9.88 |
| Vitamin B_6_ (mg/d) |  |  |  |  |  |
| Male | 2.18 ± 1.19 | 1.25 ± 0.56 | 1.73 ± 0.68 | 2.29 ± 0.72 | 3.29 ± 1.43 |
| Female | 1.58 ± 0.88 | 0.80 ± 0.34 | 1.25 ± 0.42 | 1.74 ± 0.65 | 2.45 ± 0.93 |
| Total folate (mcg/d) |  |  |  |  |  |
| Male | 448.78 ± 257.28 | 243.65 ± 96.91 | 345.43 ± 130.89 | 465.78 ± 163.97 | 703.73 ± 298.72 |
| Female | 353.80 ± 199.67 | 185.07 ± 69.17 | 281.44 ± 117.36 | 381.95 ± 138.84 | 549.14 ± 223.28 |
| Vitamin B_12_ (mcg/d) |  |  |  |  |  |
| Male | 6.08 ± 10.83 | 3.40 ± 5.98 | 4.71 ± 3.15 | 6.23 ± 6.20 | 9.52 ± 18.64 |
| Female | 4.16 ± 5.02 | 2.06 ± 1.50 | 3.01 ± 2.30 | 4.75 ± 4.79 | 6.53 ± 7.33 |
| Vitamin C (mg/d) |  |  |  |  |  |
| Male | 106.05 ± 109.95 | 49.92 ± 80.81 | 80.50 ± 79.63 | 111.22 ± 96.96 | 172.87 ± 130.60 |
| Female | 90.67 ± 95.95 | 38.89 ± 40.87 | 69.17 ± 65.91 | 93.44 ± 80.65 | 156.25 ± 128.89 |
| Vitamin E (ATE) (mg/d) |  |  |  |  |  |
| Male | 9.02 ± 8.27 | 4.92 ± 3.02 | 7.02 ± 3.48 | 9.40 ± 5.12 | 14.03 ± 13.08 |
| Female | 7.33 ± 5.82 | 4.05 ± 2.30 | 5.74 ± 3.44 | 8.15 ± 5.51 | 11.00 ± 7.70 |
| Calcium (mg/d) |  |  |  |  |  |
| Male | 960.08 ± 650.55 | 531.53 ± 287.83 | 800.69 ± 440.90 | 1008.06 ± 488.92 | 1418.75 ± 833.87 |
| Female | 755.12 ± 452.13 | 427.41 ± 230.27 | 593.24 ± 305.63 | 824.39 ± 385.69 | 1140.03 ± 485.47 |
| Magnesium (mg/d) |  |  |  |  |  |
| Male | 327.13 ± 155.93 | 190.51 ± 62.07 | 265.69 ± 64.26 | 351.65 ± 90.96 | 475.04 ± 183.23 |
| Female | 252.62 ± 116.93 | 139.22 ± 41.87 | 202.07 ± 51.40 | 277.60 ± 77.19 | 379.29 ± 113.42 |
| Zinc (mg/d) |  |  |  |  |  |
| Male | 13.87 ± 9.20 | 7.80 ± 3.16 | 11.57 ± 5.46 | 14.78 ± 7.96 | 20.17 ± 11.90 |
| Female | 9.89 ± 6.65 | 5.40 ± 2.23 | 7.82 ± 3.74 | 11.29 ± 8.23 | 14.54 ± 6.21 |
| Copper (mg/d) |  |  |  |  |  |
| Male | 1.50 ± 1.05 | 0.88 ± 0.31 | 1.21 ± 0.35 | 1.62 ± 0.84 | 2.19 ± 1.53 |
| Female | 1.16 ± 0.64 | 0.67 ± 0.22 | 0.94 ± 0.30 | 1.31 ± 0.60 | 1.69 ± 0.71 |
| Selenium (mcg/d) |  |  |  |  |  |
| Male | 124.85 ± 62.96 | 78.76 ± 30.25 | 109.18 ± 43.14 | 137.50 ± 67.55 | 165.94 ± 64.66 |
| Female | 91.21 ± 48.70 | 57.01 ± 22.74 | 80.04 ± 32.73 | 103.81 ± 41.89 | 119.88 ± 61.95 |
| Total fat (g/d) |  |  |  |  |  |
| Male | 92.18 ± 41.01 | 65.97 ± 30.63 | 88.40 ± 36.38 | 101.06 ± 40.72 | 109.35 ± 41.02 |
| Female | 67.38 ± 31.43 | 50.01 ± 21.79 | 63.03 ± 26.52 | 74.98 ± 30.98 | 80.13 ± 34.93 |
| Iron (mg/d) |  |  |  |  |  |
| Male | 17.83 ± 10.39 | 10.02 ± 3.82 | 14.28 ± 4.96 | 18.61 ± 5.79 | 26.85 ± 13.66 |
| Female | 13.30 ± 7.17 | 7.52 ± 2.95 | 10.89 ± 3.95 | 14.61 ± 5.57 | 19.54 ± 8.43 |
| Lifestyle OBS components | | |  |  |  |
| PA (MET-minute/week) | |  |  |  |  |
| Male | 1252.24 ± 1700.74 | 1129.91 ± 1804.99 | 1245.50 ± 1645.15 | 1212.19 ± 1269.82 | 1048.87 ± 1715.34 |
| Female | 836.01 ± 1115.98 | 677.37 ± 921.94 | 830.75 ± 1046.92 | 772.85 ± 880.63 | 1031.55 ± 1431.16 |
| Alcohol (g/d) |  |  |  |  |  |
| Male | 14.96 ± 32.22 | 18.76 ± 42.00 | 14.90 ± 33.12 | 15.83 ± 28.75 | 10.74 ± 23.11 |
| Female | 6.68 ± 20.30 | 6.29 ± 18.29 | 7.86 ± 25.29 | 7.33 ± 17.70 | 5.17 ± 18.93 |
| BMI (kg/m^2^) |  |  |  |  |  |
| Male | 27.80 ± 4.96 | 28.42 ± 5.31 | 27.99 ± 4.90 | 27.67 ± 4.87 | 27.20 ± 4.69 |
| Female | 27.37 ± 6.40 | 28.52 ± 6.58 | 27.63 ± 6.27 | 27.37 ± 6.50 | 26.08 ± 6.03 |
| Cotinine (ng/mL) |  |  |  |  |  |
| Male | 55.88 ± 115.11 | 91.21 ± 133.29 | 56.75 ± 119.08 | 56.22 ± 118.32 | 23.45 ± 73.92 |
| Female | 42.52 ± 98.37 | 75.01 ± 127.70 | 43.71 ± 93.75 | 37.13 ± 91.76 | 17.52 ±64.28 |
| RE, retinol equivalent; ATE, alpha-tocopherol equivalent; MET, metabolic equivalent; PA, physical activity; BMI, body mass index  ^a^ Grouped according to the quartile of OBS, and data were expressed as the means ± standard deviations | | | | | |

| Supplementary Table 2: Sensitivity analyses to evaluate the impact of individual OBS components on the OBS^a^ | | |
| --- | --- | --- |
| OBS | Male | Female |
| OBS original model 4^b^ | 0.0001 (-0.0020–0.0023) | 0.0039 (0.0014–0.0065) |
| OBS excluding total fat | -0.0002 (-0.0028–0.0019) | 0.0040 (0.0014–0.0066) |
| OBS excluding dietary fiber | 0.0004 (-0.0019–0.0027) | 0.0042 (0.0016–0.0067) |
| OBS excluding carotene | 0.0000 (-0.0021–0.0022) | 0.0046 (0.0016–0.0065) |
| OBS excluding riboflavin | -0.0002 (-0.0022–0.0025) | 0.0044 (0.0017–0.0071) |
| OBS excluding niacin | 0.0003 (-0.0021–0.0026) | 0.0040 (0.0012–0.0069) |
| OBS excluding vitamin B_6_ | 0.0001 (-0.0022–0.0024) | 0.0043 (0.0017–0.0040) |
| OBS excluding total folate | 0.0001 (-0.0022–0.0023) | 0.0041 (0.0014–0.0068) |
| OBS excluding vitamin B_12_ | 0.0002 (-0.0021–0.0025) | 0.0039 (0.0013–0.0066) |
| OBS excluding vitamin C | -0.0001 (-0.0022–0.0019) | 0.0043 (0.0016–0.0069) |
| OBS excluding vitamin E | 0.0004 (-0.0018–0.0026) | 0.0042 (0.0015–0.0069) |
| OBS excluding calcium | 0.0003 (-0.0020–0.0025) | 0.0045 (0.0017–0.0073) |
| OBS excluding magnesium | 0.0001 (-0.0022–0.0025) | 0.0043 (0.0014–0.0071) |
| OBS excluding iron | 0.0000 (-0.0019–0.0020) | 0.0037 (0.0013–0.0060) |
| OBS excluding zinc | 0.0002 (-0.0020–0.0024) | 0.0041 (0.0014–0.0068) |
| OBS excluding copper | 0.0001 (-0.0021–0.0023) | 0.0043 (0.0015–0.0070) |
| OBS excluding selenium | 0.0004 (-0.0019–0.0026) | 0.0043 (0.0014–0.0072) |
| OBS excluding alcohol | 0.0006 (-0.0017–0.0029) | 0.0040 (0.0014–0.0067) |
| OBS excluding body mass index | -0.0003 (-0.0024–0.0019) | 0.0038 (0.0014–0.0062) |
| OBS excluding cotinine | 0.0003 (-0.0020–0.0026) | 0.0038 (0.0015–0.0062) |
| OBS excluding physical activity | -0.0003 (-0.0026–0.0020) | 0.0037 (0.0010–0.0064) |
| ^a^ Grouped according to the quartile of OBS, and data were expressed as the beta estimates and its 95% confidence intervals  ^b^ Adjusting for age, ethnicity, education, poverty index, dietary energy intake, and CRP | | |
